# The uterine corpus flexion angle: a novel and objective sonographic marker for endometriosis severity and the quantification of the question mark sign

**DOI:** 10.1186/s12905-026-04695-6

**Published:** 2026-07-25

**Authors:** Jing Chen, Shulin Zhong, Jiale Liang, Fang Liu, Lijuan Li, Shuyi Guo, Xiaoling Leng

**Affiliations:** 1 Department of Ultrasonography, The Tenth Affiliated Hospital, Southern Medical University (Dongguan People’s Hospital), Dongguan, Guangdong China; 2 Shenzhen School of Clinical Medicine, Southern Medical University, Shenzhen, China; 3 Department of Gynecology, The Tenth Affiliated Hospital, Southern Medical University (Dongguan People’s Hospital), Dongguan, Guangdong China

**Keywords:** Endometriosis, Pelvic adhesions, Ultrasonography, Uterine corpus flexion angle, Uterine flexion angle, Question mark sign

## Abstract

**Background:**

This study aimed to investigate the correlation between the uterine corpus flexion angle (UCFA) and the severity of endometriosis, evaluate its clinical utility as a novel objective sonographic parameter, and provide an angular definition for the question mark sign.

**Methods:**

This mixed prospective-retrospective case–control study enrolled participants in a 1:1:1 ratio into three groups: the endometriosis group, the benign control group, and the normal control group. All participants underwent transvaginal ultrasonography (TVUS). The UCFA and uterine flexion angle (UFA) were independently measured in the standard midsagittal plane by two blinded sonographers. The subjective question mark sign was assessed by sonographers. In the endometriosis group, pelvic adhesions were graded intraoperatively using the revised American Society for Reproductive Medicine (rASRM) criteria. The correlation of the UCFA with the severity of endometriosis and the question mark sign was evaluated via Spearman’s correlation, multivariate linear regression, and receiver operating characteristic curve analyses.

**Results:**

The UCFA progressively increased with higher rASRM stage (*P* < 0.001) and greater pelvic adhesion severity (*P* < 0.001). Multivariate linear regression analysis revealed that UCFA was primarily determined by pelvic adhesion score (*β* = 0.441), whereas UFA was predominantly influenced by baseline uterine position (*β* = 0.508). Subgroup analysis in 166 adenomyosis-free endometriosis patients confirmed that adhesion score remained the primary predictor of UCFA (*β* = 0.457). The optimal cutoff for the sonographic question mark sign was UCFA ≥ 220°, with an area under the curve (AUC) of 0.958. UCFA values were relatively stable in women without endometriosis, with a median of 179.0° in the benign control group and 178.0° in the normal group.

**Conclusions:**

The UCFA is a reliable objective sonographic marker for assessing the severity of endometriosis-related pelvic adhesions. The proposed criterion of UCFA ≥ 220° provides a validated, quantitative definition for the question mark sign, offering a practical tool for standardized ultrasonography assessment and preoperative surgical risk stratification.

**Supplementary Information:**

The online version contains supplementary material available at https://doi.org/10.1186/s12905-026-04695-6.

## Background

Endometriosis is a systemic inflammatory condition associated with the presence of displaced endometrium-like tissues outside the uterus [1]. As a common benign gynecological disease, it is often overlooked [2]. Its diagnosis is often delayed, which has an average time of 5–12 years after symptom onset, most women consult 3 or more clinicians prior to diagnosis [3]. The main clinical manifestations include menstrual pain, dyspareunia, lower abdominal pain, and subfertility or infertility [4, 5].

A retroflexed uterus is defined as a condition in which the cervix is pointing downward and forward, and the uterine corpus axis is directed backward toward the sacral hollow [6]. The retroflexed uterus is a normal physiologic variant observed in 20% of women. However, it can also be associated with chronic retracting posterior deep infiltrating endometriosis and adhesions [7]. Fixed uterine retroflexion, particularly the question mark sign, is one of the indirect findings of endometriosis [8]. This indicates significant adhesions pulling the uterine fundus or corpus backward to the bowel or the posterior pelvic wall [9].

This prompted the hypothesis that the uterine corpus flexion angle (UCFA), rather than the uterine flexion angle (UFA), is pathologically correlated with endometriosis. To test the hypothesis, this study aimed to systematically evaluate the correlation of both UCFA and UFA with the presence and severity of endometriosis and to establish a new objective and precise ultrasonographic parameter for clinical assessment.

## Methods

### Study design and participants

This was a mixed prospective-retrospective observational case–control study conducted at The Tenth Affiliated Hospital of Southern Medical University (Dongguan People's Hospital) between March 2023 and June 2025. The study was approved by the Hospital Ethics Committee (registration number: KYKT2023-039) and performed in accordance with the Declaration of Helsinki.

Data from 207 patients with endometriosis were collected prospectively, while 207 patients each in the benign control group and normal control group were enrolled retrospectively during the identical study period. Written informed consent was obtained from all patients with endometriosis. Informed consent was waived for the two control groups by the ethics committee due to the retrospective and anonymized nature of data analysis, which involved no additional interventions and did not affect routine clinical care.

### Inclusion criteria

The endometriosis group comprised patients who underwent laparoscopic surgery for clinically suspected endometriosis, with the diagnosis subsequently confirmed by postoperative histopathological examination.

The benign control group included patients who underwent laparoscopic surgery for sonographically identified benign ovarian cystic lesions, including mature teratomas and serous or mucinous cystadenomas. Intraoperative and final pathological examinations confirmed the benign nature of these lesions and ruled out visible pelvic endometriosis.

The normal control group consisted of participants who underwent routine physical examination, with no abnormal findings on gynecological examination and transvaginal ultrasound (TVUS).

### Exclusion criteria

All participants were excluded if they had: suspected or confirmed gynecological malignancy; age < 18 years or postmenopausal status; current pregnancy; uterine leiomyomas with maximum diameter > 3 cm; congenital uterine anomalies; current use of an intrauterine device; adenomyosis (excluded only from the benign and normal control groups to eliminate confounding).

All patients underwent TVUS performed by expert sonographers with at least 10 years of clinical experience and systematic training in the International Deep Endometriosis Analysis (IDEA) protocol, using an endovaginal probe. All surgical procedures were performed by the same dedicated gynecologic team specialized in the diagnosis and treatment of endometriosis.

Sample size was calculated based on a pilot study demonstrating a mean difference of 25° in uterine corpus flexion angle (UCFA) between the endometriosis and benign control groups (standard deviation = 40°). With a two-sided significance level of 0.05 and statistical power of 90%, a minimum of 170 participants per group was required to detect a statistically significant difference. Given the three-group study design, we adopted a 1:1:1 concurrent enrollment strategy. Based on the 207 prospectively enrolled patients in the endometriosis group, 207 patients were consecutively enrolled in each of the two control groups during the same study period.

### Transvaginal ultrasound examination

All TVUS examinations were performed using endovaginal probes (4–9 MHz, Voluson E6, GE Healthcare, Zipf, Austria; 6–12 MHz, Resona R9, Mindray, Shenzhen, China) by senior sonographers with ≥ 10 years of clinical experience and systematic training in the International Deep Endometriosis Analysis (IDEA) protocol. Patients in the endometriosis group underwent a complete four-step IDEA protocol examination within 1 month before surgery. Patients in the benign and normal control groups underwent routine gynecological TVUS.

Adenomyosis was diagnosed on TVUS according to the MUSA criteria [10], including myometrial asymmetry, heterogeneous myometrial echotexture, myometrial cysts, indistinct endometrial–myometrial junction, and fan-shaped shadowing.

All participants were instructed to empty their bladder before examination and placed in the lithotomy position. The probe was inserted gently to avoid external force that might alter natural uterine position. The standard midsagittal plane was defined as the plane clearly visualizing the entire length of the cervix and uterine corpus. A clear static image of this plane was acquired and stored for subsequent analysis.

The uterus was classified as anteverted if the uterine corpus was oriented toward the anterior abdominal wall or retroverted if oriented toward the posterior pelvic wall. A clear image of the uterine midsagittal plane was acquired and stored for subsequent analysis.

### Angle definition and measurement protocol

Uterine flexion angle (UFA): The anterior angle between the longitudinal axis of the cervix and the longitudinal axis of the uterine corpus [11].

Uterine corpus flexion angle (UCFA): The anterior angle at the midpoint of the uterine corpus curvature, formed between the axis of the upper uterine segment and the axis of the lower uterine segment. This angle quantifies the curvature of the uterine corpus.

**Fig. 1:**
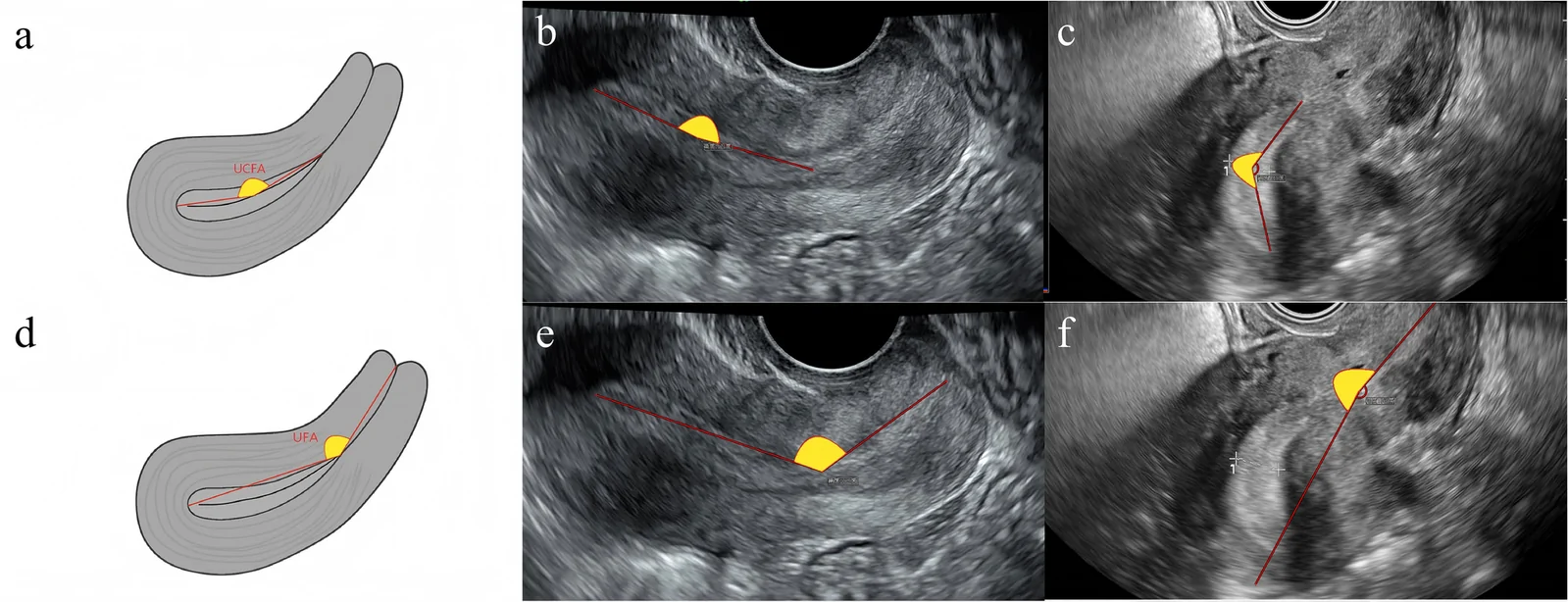
Schematic and ultrasonographic representations of uterine corpus flexion angle (UCFA) and uterine flexion angle (UFA). **a** Schematic diagram of UCFA measurement. The vertex of the angle is located at the midpoint of the endometrial line of the uterine corpus, and the anterior angle is formed by the anatomical long axis of the upper uterine segment (from the midpoint to the fundal endometrial apex) and the long axis of the lower uterine segment (from the midpoint to the cervico-corporeal junction). **b **Ultrasonographic image of UCFA measurement in an anteverted uterus with mild intrinsic curvature of the uterine corpus. **c** Ultrasonographic image of UCFA measurement in a patient with severe endometriosis and a positive question mark sign. **d** Schematic diagram of the UFA measurement. **e** Ultrasonographic image of UFA measurement in an anteverted uterus. **f** Ultrasonographic image of UFA measurement in a patient with severe endometriosis

Angle measurements were performed independently by two sonographers who were blinded to the clinical and surgical data, using the angle measurement tool within our institution’s Picture Archiving and Communication System (PACS) on the stored midsagittal uterine images (Fig. 1). All UCFA measurements were performed strictly following the standardized protocol developed in this study (Supplementary File 1).

Inter-rater reliability was assessed by duplicate measurements in 52 randomly selected cases. The single-measure intraclass correlation coefficient (ICC(2,1)) using a two-way random-effects model was 0.972 (95% CI: 0.952–0.984), demonstrating excellent reliability.

Assessment of the question mark sign: The presence of the question mark sign was jointly assessed by two sonographers. In cases of disagreement, a third sonographer provided an independent assessment, and the majority opinion was adopted as the final determination.

### Surgical assessment and adhesion scoring

All laparoscopic surgeries for both the endometriosis group and the benign control group were performed exclusively by a core team of 4 dedicated gynecologic endometriosis surgeons with over 10 years of specialized experience in the diagnosis and treatment of endometriosis.

Endometriosis group: The surgeons assessed and staged the condition based on intraoperative observations, strictly following the revised American Society for Reproductive Medicine adhesion scoring scale. Endometriosis was then staged as mild (1–15 points), moderate (16–40 points), or severe (> 40 points). Pelvic adhesions were investigated using a modified ASRM scoring system. Adhesions were classified as mild (1–2 points), moderate (3–4 points), or severe (5–6 points). Severe adhesions were defined as dense adhesive tissues involving > 25% of the operative field. The presence of dense uterine fixation to the rectum and complete obliteration of the pouch of Douglas (POD) were specifically documented.

Benign control group: Pelvic adhesion scores for the benign control cohort were assigned by the original operating surgeons themselves based on their own standardized operative notes, supplemented by available intraoperative images and videos for some cases. Scoring was strictly performed using the identical modified ASRM adhesion scoring system applied to the endometriosis group to ensure uniformity of assessment criteria between the two groups.

### Statistical analysis

The participants were stratified according to uterine position (anteverted/retroverted) for the subgroup analyses. The analysis of qualitative variables involved calculating the absolute and percentage frequencies of all possible values that these variables could take. Continuous data with non-normal distributions were presented as median (interquartile range, IQR). The 95% confidence intervals for the median were estimated using the bootstrap method with 1,000 replications to provide robust estimates. The correlations between quantitative variables were analyzed using the Spearman’s correlation coefficient. The comparison of quantitative variables between two groups was conducted using the Mann–Whitney U test. To compare quantitative variables across three or more groups, the Kruskal–Wallis test was utilized, followed by the Dunn’s post-hoc test in the presence of statistically significant differences among the groups. Categorical data were expressed as numbers (percentages), and group comparisons were conducted using the chi-square (*χ*^2^) test.

A two-sided *P*-value of < 0.05 indicated statistically significant differences. In the endometriosis group, variables associated with the flexion angles at *P* < 0.10 in the univariate analyses were considered candidates for the multivariate model.

The diagnostic performance of the UCFA was evaluated via receiver operating characteristic (ROC) curve analysis. The area under the curve (AUC) and optimal cutoff values were calculated.

The statistical analyses were performed using the Statistical Package for the Social Sciences software (version 26.0; IBM Corp., Armonk, NY, USA).

## Results

### Clinical characteristics of the participants

In total, 621 women were included in the final analysis. Gravidity, parity, history of uterine corpus surgery, and uterine position were comparable among the three groups. The maximum diameter of ovarian cystic lesions was also similar between the endometriosis and benign control groups. The endometriosis group had a significantly lower body mass index (BMI) than the benign control and normal groups. The normal group was significantly younger than the benign control and endometriosis groups (Table 1).

**Table 1:** Clinical characteristics of the three patient groups

Variables	Endometriosis (*n* = 207)	Benign (*n* = 207)	Normal (*n* = 207)	*P* Value
Age, median (IQR)	33.0 (28.0, 42.0)	37.0 (32.0, 42.0)	34.0 (29.0, 39.0)	< 0.001
BMI, median (IQR)	21.6 (20.0, 23.4)	22.7 (20.2, 24.7)	22.1 (19.8, 24.1)	0.028
Gravidity [*n* (%)]				0.395
0	48 (23.2)	57 (27.5)	53(25.6)	
1	50 (24.1)	41 (19.8)	35 (16.9)	
≥ 2	109(52.7)	109(52.7)	119(57.5)	
Parity [*n* (%)]				0.948
0	64(30.9)	67 (32.4)	61(29.5)	
1	61 (29.5)	63 (30.4)	61 (29.5)	
≥ 2	82(39.6)	77(37.2)	85(41.1)	
History of uterine corpus surgery (cesarean section, myomectomy)				0.451
0	151(72.9)	157(75.8)	162(78.3)	
1	56(27.1)	50(24.2)	45(21.7)	
Uterine position [*n* (%)]				0.514
Anteverted	139 (68.6)	145(70.0)	134 (64.7)	
Retrovered	68 (32.9)	62 (30.0)	73 (35.3)	
Maximum diameter, median (IQR)	58.0 (45.0, 76.5)	61.0 (46.5, 74.5)	——	0.470

Continuous variables are presented as median (interquartile range, IQR) and compared using the Kruskal–Wallis test. Categorical variables are presented as *n* (%) and compared using the *χ*^2^ test. The "Maximum diameter" refers to the maximum diameter of ovarian cysts in endometriosis and benign group, which is not applicable to the normal control group

### Two angle measurements across the uterine position subgroups among the three patient groups

Stratified by uterine position, UCFA was consistently and significantly higher in the endometriosis group than in both control groups, regardless of whether the uterus was anteverted or retroverted (all *P* < 0.001). In contrast, UFA differed significantly across groups only in the anteverted subgroup, with no significant difference observed in the retroverted subgroup (Table 2). Therefore, compared with the UFA, the UCFA is more suitable for assessing endometriosis and pelvic adhesions.The median UCFA was remarkably similar between the benign control and normal groups (179.0° vs 178.0°), with fully overlapping 95% confidence intervals for the median (177.0º–180.0° for both groups) as estimated using 1,000 replications bootstrap resampling. The distributions in both groups exhibited comparable dispersion (IQR: 16° vs. 15°). Based on these results, the presence of ovarian cysts did not significantly affect the UCFA. In the absence of space-occupying lesions of the uterine corpus/cavity and severe pelvic adhesions, the uterine corpus flexion angle remains relatively stable.

**Table 2:** Comparison of UFA and UCFA across the three study groups, stratified by uterine position

Uterine Position	Group	*n*	UFA (°), median (IQR)	*Pairwise P values*	UCFA (°), median (IQR)	*Pairwise P values*
Anteverted uterus	Endometriosis group	142	165.0 (146.8, 185.3)	P* < 0.001	185.5 (175.8, 210.3)	P* < 0.001
	Benign control group	145	154.0 (139.0, 168.0)	*P*^*#*^ < 0.001	176.0 (171.0, 182.5)	*P*^*#*^ < 0.001
	Normal control group	134	151.0 (139.0, 162.0)	*P*^*&*^ = *0.170*	176.0 (170.0, 182.3)	*P*^*&*^ = *0.408*
Retroverted uterus	Endometriosis group	65	219.0 (204.0, 233.5)	P* = *0.917*	220.0 (196.0, 233.5)	P* < 0.001
	Benign control group	62	217.0 (210.0, 227.0)	*P*^*#*^ = *0.136*	184.5 (178.0, 193.3)	*P*^*#*^ < 0.001
	Normal control group	73	219.0 (208.5, 245.0)	*P*^*&*^ = *0.127*	185.0 (178.0, 195.0)	*P*^*&*^ = *0.632*

*Abbreviations UFA* Uterine flexion angle, *UCFA* Uterine corpus flexion angle, *IQR* Interquartile range

P*: *P* value for comparison between the endometriosis group and benign control group

*P*^*#*^: *P* value for comparison between the endometriosis group and normal control group

*P*^*&*^: *P* value for comparison between the benign control group and normal control group

### Correlation between the UCFA and the clinical pathological features of endometriosis

The UCFA progressively increased with disease severity regardless of uterine position (Table 3). Both the anteverted and retroverted uteri showed significant positive correlations between pelvic adhesion severity and UCFA (all *P* < 0.001). Deep infiltrating endometriosis was correlated with a higher overall UCFA (207° vs. 188°, *P* = 0.011), a difference primarily observed in patients with anteverted uteri. No significant difference was found in the retroverted uteri (*P* = 0.346). Adenomyosis was significantly associated with increased uterine retroversion (219° vs. 188.5°, *P* < 0.001).

**Table 3:** Comparison of the uterine corpus flexion angle (UCFA) by uterine position subgroups in Endometriosis group

Clinical features	Category	UCFA (°), median (IQR) [n]			*P* Value (overall)	*P* Value (anteverted)	*P* Value (retroverted)
		**All patients** **(*n* = 207)**	**Anteverted uterus** **(*n* = 139)**	**Retroverted uterus (*n* = 68)**			
rASRM Stage	Mild	184.0 (179.0–188.8) [16]	182.0 (179.0–188.0) [11]	188.0 (181.5–220.5) [5]	< 0.001	0.003	< 0.001
	Moderate	183.0 (177.0–194.0)[71]	179.0 (175.0–190.8)[52]	191.0 (181.0–211.0)[19]			
	Severe	216.0 (186.5–232.8)[120]	202.0 (176.0–229.0)[76]	228.0 (212.3–241.0)[44]			
Pelvic Adhesion Score	Mild	179.0 (173.5–186.0)[53]	178.0 (171.0–184.0)[39]	185.5 (182.0–194.8) [14]	< 0.001	< 0.001	< 0.001
	Moderate	196.0 (179.0–219.3)[78]	188.0 (176.8–206.3)[54]	213.5 (192.8–228.0)[24]			
	Severe	223.0 (199.5–239.0)[76]	207.5 (180.5–232.8)[46]	231.5 (218.0–247.8)[30]			
DIE	Absent	188.0 (177.0–222.0)[121]	182.0 (174.0–200.5)[84]	216.0 (189.5–232.0)[37]	0.011	0.024	0.346
	Present	207.0 (180.8–229.0)[86]	201.0 (177.0–223.0)[55]	221.0 (199.0–234.0)[31]			
Adenomyosis	Absent	188.5 (177.0–222.3)[166]	183.0 (175.0–206.5) [117]	210.0 (186.0–228.0) [49]	< 0.001	0.004	0.025
	Present	219.0 (196.5–238.0)[41]	206.5 (184.0–223.0) [22]	228.0 (217.0–243.5) [19]			

Data are presented as median (interquartile range, IQR) with sample size in brackets. Kruskal–Wallis test was used for between-group comparisons

*rASRM* revised American Society for Reproductive Medicine, *DIE* Deep infiltrating endometriosis

Spearman’s rank correlation analysis revealed a statistically significant but extremely weak positive correlation between UCFA and disease duration (rs = 0.152, *P* = 0.029).

UCFA was significantly higher in patients with dysmenorrhea than in those without (Z = − 2.787, *P* = 0.005). No significant difference in UCFA was found between patients with and without dyspareunia (Z = − 1.139, *P* = 0.255), likely due to the small sample size of the dyspareunia subgroup. No significant associations were observed between UFA and either dysmenorrhea or dyspareunia.

### Analysis of the multiple linear regression models for the UFA and UCFA

Based on the univariate correlation and multivariate linear regression analyses, maximum cyst diameter (*r* = 0.061, *P* = 0.193) and history of uterine surgery (*r* = 0.040, *P* = 0.286) were not significantly associated with the UCFA. Thus, the final model retained uterine position, rASRM stage, pelvic adhesion score, and adenomyosis (Table 4).

**Table 4:** Univariate analysis of the UFA and UCFA in the endometriosis group

	**Variables**	***n* = 139(A)**		***n* = 68(R)**	
		**ρ/Z**	***P* Value**	**ρ/Z**	***P* Value**
UFA	rASRM	0.021	0.809	0.215	0.078
	Adhesion	0.245	0.004	0.149	0.227
	Adenomyosis	0.239	0.005	−0.032	0.456
UCFA	rASRM	0.276	0.001	0.557	< 0.001
	Adhesion	0.445	< 0.001	0.569	< 0.001
	Adenomyosis	0.244	0.004	0.337	0.005

Spearman correlation analysis was performed, with *ρ* representing the correlation coefficient and *Z* representing the test statistic

*Group A* Anteverted uterus (*n* = 139), *Group R* Retroverted uterus (*n* = 68), *rASRM* revised American Society for Reproductive Medicine stage, Adhesion, pelvic adhesion score

*P* < 0.05 was considered statistically significant

According to the multivariate linear regression analysis, both the UFA and UCFA were statistically significant in their respective models. The UCFA model exhibited a stronger explanatory power (adjusted *R*^2^ = 0.341) compared with the UFA model (adjusted *R*^2^ = 0.293). The pelvic adhesion score was a significantly stronger independent predictor of UCFA (standardized *β* = 0.441, *P* < 0.001) than of UFA (standardized *β* = 0.132, *P* = 0.030). In contrast, uterine position was the dominant factor influencing UFA (*β* = 0.508, *P* < 0.001), exerting a comparatively weaker effect on UCFA (*β* = 0.249, *P* < 0.001). Furthermore, the presence of adenomyosis was independently associated with UCFA (*P* = 0.008) but not with UFA (*P* = 0.406) (Table 5).

**Table 5:** Comparative multivariate linear regression analysis of the determinants for the two angles

**Predictor Variable**	**UCFA Model**		**UFA Model**	
	***B* (95% CI)**	**Standardized* β* (*P*)**	***B* (95% CI)**	**Standardized *β* (*P*)**
Model Characteristics				
Adjusted *R*^2^	0.341		0.293	
Model *P* value	< 0.001		< 0.001	
Regression Coefficients				
Constant	153.86 (143.19, 164.53)	- (< 0.001)	114.33 (98.53, 130.12)	- (< 0.001)
Adhesion Score	12.90 (9.54, 16.26)	0.441 (< 0.001)	5.53 (0.55, 10.51)	0.132 (0.030)
Retroverted vs. Anteverted	14.19 (7.74, 20.64)	0.249 (< 0.001)	41.39 (31.84, 50.94)	0.508 (< 0.001)
Adenomyosis (Yes vs. No)	10.59 (2.84, 18.35)	0.158 (0.008)	4.86 (−6.63, 16.34)	0.051 (0.406)

*Abbreviations B*, Unstandardized regression coefficient, *CI* Confidence interval, *β* Standardized regression coefficient

All variance inflation factors (VIFs) were < 1.1, indicating no multicollinearity

### Subgroup analysis in endometriosis patients without adenomyosis

To eliminate the potential confounding effect of adenomyosis on uterine anatomy, we performed a subgroup analysis within the endometriosis group restricted to patients without adenomyosis (*n* = 166). In this subgroup, the UCFA still increased progressively and significantly with ascending rASRM stage (*H* = 21.274, df = 2, *P* < 0.001) and worsening pelvic adhesion severity (*H* = 32.798, df = 2, *P* < 0.001).

Multivariate linear regression confirmed that pelvic adhesion score remained the strongest independent predictor of UCFA (standardized *β* = 0.457, *P* < 0.001), followed by uterine position (standardized *β* = 0.246, *P* < 0.001). The adjusted *R*^*2*^ of the model was 0.279, consistent with the full endometriosis cohort.

These findings confirm that the association between UCFA and endometriosis-related posterior pelvic compartment adhesions is independent of adenomyosis and further validate the reliability of UCFA as an objective sonographic marker.

### Question mark sign

In the endometriosis group, 50 women were diagnosed with the question mark sign on ultrasound. It had a high specificity (87.0%) but limited sensitivity (43.4%) for severe pelvic adhesions. The ROC analysis showed that the UCFA effectively discriminated the question mark sign, with an AUC of 0.958 (95% CI: 0.934–0.982, *P* < 0.001). The maximum Youden index was 0.801, yielding an optimal cutoff of UCFA ≥ 220°, corresponding to a sensitivity of 91.3% and a specificity of 88.8%. These results provide a reliable and reproducible quantitative criterion for this classic yet subjective ultrasound sign.

## Discussion

This study showed that UCFA was a novel, objective sonographic marker that quantified the anatomical distortion associated with endometriosis. Our findings confirmed that UCFA is a quantifiable parameter strongly correlated with the pathological features of endometriosis, particularly pelvic adhesions. Further, the UFA is primarily influenced by uterine version.

The multivariate linear regression analysis validated our hypothesis that the dominant influence of pelvic adhesion scores on UCFA (*β* = 0.441) supported the model of posterior compartment disease that exerts direct traction on the uterine corpus. The UFA was primarily determined by uterine position (*β* = 0.508), with only a modest additional effect from adhesions (*β* = 0.132).

In 2015, Di Donato et al. initially described the question mark sign as a sonographic feature indicative of adenomyosis. If the uterine corpus was flexed backward, the uterine fundus was facing the posterior pelvic compartment, and the cervix was directed anteriorly toward the urinary bladder [12]. Further, it is recognized as an indirect sonographic indicator of endometriosis and a marker of pelvic adhesions [8, 9]. Adenomyosis emerged as the third independent determinant of UCFA (*β* = 0.158). However, it had no significant independent effect on UFA. Multivariate regression analysis confirmed that both pelvic adhesion score (standardized *β* = 0.441) and adenomyosis (standardized *β* = 0.158) are independent predictors of increased UCFA. Therefore, a significantly elevated UCFA in clinical practice warrants consideration of both pathological conditions. These two factors may act synergistically to exacerbate uterine retroflexion, jointly contributing to the pathological basis of the question mark sign.

Not all cases of endometriosis are accompanied by adenomyosis. To exclude the potential confounding effect of adenomyosis and verify the robustness of our findings, we performed a subgroup analysis restricted to endometriosis patients without adenomyosis (n = 166). The results confirmed that pelvic adhesion score remained the strongest independent predictor of UCFA, and the association between UCFA and endometriosis severity was consistent and robust, supporting the finding that UCFA primarily reflects adhesion-related uterine distortion, even though adenomyosis may also contribute to an increased UCFA.

The extremely weak correlation between UCFA and disease duration can be explained by two factors. First, given the insidious onset and widespread diagnostic delay of endometriosis, disease duration based on patients’ self-reported symptom onset or initial diagnosis is inherently susceptible to recall bias. Second, the severity of pelvic adhesions shows no definitive association with the length of disease course.

In both the normal and benign control groups, the UCFA remained relatively stable, which can be attributed to the synergistic combination of the inherent structural resilience of the uterus as a muscular organ and its physiological contractile tone.

Via the ROC curve analysis, the optimal cutoff value for the question mark sign was determined to be 220°. Compared with the subjective assessment of the question mark sign, this provides a clear, measurable diagnostic threshold for sonographers and can improve interobserver consistency. In routine gynecologic ultrasound examinations, UCFA can be a practical tool for the rapid screening of endometriosis-associated severe adhesions. The presence of the question mark sign or a significantly elevated UCFA indicates dense posterior pelvic adhesions, which may require the involvement of colorectal or urologic surgeons and is associated with a longer surgical duration and higher complication risks [13].

Consistent with previous literature, the endometriosis group had a significantly lower BMI than the control groups [14], but this difference did not confound the independent association between UCFA and pelvic adhesion severity. Potential explanations for this finding include chronic pain-induced appetite suppression, gastrointestinal side effects of non-steroidal anti-inflammatory drugs [15], and the complex interplay between body composition and chronic inflammation in endometriosis pathophysiology [16].

By defining a clear anatomical landmark and a standardized measurement protocol in the standard midsagittal plane, we have established UCFA as an objective, quantitative parameter that converts subjective impression into a numerical value. Our data confirmed excellent inter-rater reliability for UCFA measurement (ICC = 0.972), demonstrating that this standardized approach can be consistently applied across different operators.A standardized measurement protocol also supports future development of automated and artificial intelligence (AI)-assisted ultrasound analysis. AI algorithms require large datasets of standardized, reproducible measurements with well-defined anatomical reference points for training and validation. The UCFA measurement protocol, with its clear image acquisition requirements and objective calculation method, provides an ideal training target for AI models.

Clinically, the strength of adhesions is the most significant factor determining surgical time or complexity [17]. Although a normal UCFA cannot fully exclude pelvic adhesions, a distinctly elevated UCFA serves as a convenient, easily detected warning sign for potential posterior compartment distortion and increased surgical complexity. As a simple, rapid indicator, UCFA can be integrated into routine gynecologic ultrasound to enhance preoperative risk stratification. When combined with other classic sonographic features of endometriosis such as the sliding sign and kissing sign, UCFA improves the overall reliability of preoperative assessment and helps surgeons better anticipate surgical difficulty.

This study has several limitations. First, the single-center study design may restrict the generalizability of our findings. Second, the mixed prospective-retrospective design, with prospective data collection for the endometriosis group and retrospective enrollment for the two control groups, carries a potential risk of selection bias. Third, the predictive value of the UCFA ≥ 220° cutoff for surgical complexity requires further validation. Future large-scale prospective multicenter studies are warranted to confirm the clinical utility of UCFA in assessing endometriosis-related pelvic adhesions and surgical risk.

## Conclusions

The UCFA is a robust and objective ultrasonographic parameter that is closely correlated with the severity of endometriosis-associated pelvic adhesions. The proposed cutoff of UCFA ≥ 220° transforms the qualitative question mark sign into a quantitative diagnostic tool that can provides a quantitative reference for widespread clinical application. Clinically, an elevated UCFA serves as a clear red flag for dense posterior pelvic adhesions, alerting surgeons to the need for preoperative bowel preparation, colorectal/urologic consultation, and appropriate patient counseling regarding surgical risks. Furthermore, the highly reproducible UCFA measurement with clear anatomical reference points provides a reliable standardized training target for artificial intelligence-assisted diagnostic models, facilitating the development of intelligent diagnostic systems for endometriosis.

## Supplementary Information

Supplementary Material 1

## Data Availability

The datasets used and/or analysed during the current study are available from the corresponding author on reasonable request.

## Abbreviations

UFA: Uterine flexion angle
UCFA: Uterine corpus flexion angle
DIE: Deep infiltrating endometriosis
BMI: Body mass index
TVUS: Transvaginal ultrasonography
rASRM: Revised American Society for Reproductive Medicine
ICC: Intraclass correlation coefficient
ROC: Receiver operating characteristic
AUC: Area under the curve
CI: Confidence interval
IQR: Interquartile range
POD: Pouch of Douglas
