# Supplementary material for: The uterine corpus flexion angle: a novel and objective sonographic marker for endometriosis severity and the quantification of the question mark sign
Source: BMC Womens Health. 2026 Jul 25;26:543. doi: 10.1186/s12905-026-04695-6 (PMC13685137; doi:10.1186/s12905-026-04695-6)
Supplement: Supplementary file 1 [file 12905_2026_4695_MOESM1_ESM.docx]

Supplementary File 1:Standardized Measurement Protocol for Uterine Corpus Flexion Angle (UCFA)

**Introduction**

This document presents the standardized measurement protocol for uterine corpus flexion angle (UCFA), which was originally developed for the present study. The protocol covers the anatomical definition of UCFA, the standardized three-point measurement procedure, and the quantitative cutoff criterion for the sonographic question mark sign.

For completeness of reproduction, the measurement method of uterine flexion angle (UFA) , adopted from previously published literature (reference [11] in the main manuscript) , is also briefly described here.

All angle measurements were performed on static midsagittal transvaginal ultrasound images, using the built-in angle tool of the clinical Picture Archiving and Communication System (PACS).Alternatively, angle measurements can also be performed directly on the ultrasound system using its built-in angle measurement tool.

**Patient Preparation and Examination Conditions**

(1) Bladder status: Patients are instructed to completely empty the bladder before examination, to avoid uterine traction and displacement induced by bladder filling, which would alter the natural anatomical morphology of the uterus.
(2) Patient position: All patients placed in the lithotomy position.
(3) Ultrasound equipment: Endovaginal probes are used for scanning.
(4) Probe operation specification: The vaginal probe is gently inserted and only lightly contacts the cervix. Excessive compression or manual displacement of the uterus is strictly prohibited, to fully preserve the natural contour and intrinsic curvature of the uterine corpus.

**Standard Uterine Midsagittal Plane**

A standard midsagittal plane is confirmed when a continuous and complete endometrial echo line is visible along the long axis of the uterus from the cervix to the fundus. All angle measurements must be performed on the standard uterine midsagittal plane.

**Definitions of Angles**

1. Uterine Corpus Flexion Angle (UCFA)

The UCFA takes the midpoint of the endometrial line of the uterine corpus as its vertex, and is formed by the axis of the upper uterine segment (from the midpoint to the fundus) and the axis of the lower uterine segment (from the midpoint to the cervico-corporeal junction). This indicator independently quantifies the intrinsic curvature of the uterine corpus itself, independent of the overall cervico-corporeal flexion status.This anatomical definition was originally developed for the present study.

2.Uterine Flexion Angle (UFA)

The UFA is defined as the anterior angle formed by the longitudinal axis of the cervix and the longitudinal axis of the uterine corpus.This definition and measurement standard were adopted from previously published literature (Ref. [11]).

**Step-by-Step Measurement Procedure on PACS System**

1. Measurement for Uterine Corpus Flexion Angle (UCFA) (Standard three-point Method,Figure 1a-c)

(1) Confirm the standard midsagittal plane, in which the endometrial echo line of the uterine corpus is continuous and clearly displayed.

(2) Locate three anatomical landmark points along the midline of the endometrium:

Vertex point: the vertex of the angle is placed at the midpoint of the endometrial line of the uterine corpus by visual estimation.

Distal point 1: the apex of the endometrial echo at the uterine fundus.

Distal point 2: the cervico-corporeal junction at the isthmic level.

(3)First straight line: connect the vertex point to the fundal endometrial apex, representing the anatomical long axis of the upper uterine segment.

(4)Second straight line: connect the vertex point to the cervico-corporeal junction, representing the anatomical long axis of the lower uterine segment.

(5)Measure and record the value of the anterior angle formed by the two straight lines.

2. Measurement Steps for Uterine Flexion Angle (UFA)

Angle of uterine flexion was evaluated as the anterior angle between the longitudinal axis of the cervix and the longitudinal axis of uterine corpus(Figure 1d-f).

**Exclusion Criteria for Accurate Measurement**

Under the following conditions, measurement of the uterine corpus flexion angle is not recommended, and the interpretation of measurement results should be made with caution:

1.Uterine leiomyomas with a maximum diameter > 3 cm, which distort the anatomical contour of the uterine corpus.

Figure 1. Schematic and ultrasonographic representations of Uterine corpus flexion angle (UCFA) and Uterine flexion angle (UFA) .(a) Schematic diagram of UCFA measurement. The vertex of the angle is located at the midpoint of the endometrial line of the uterine corpus, and the anterior angle is formed by the anatomical long axis of the upper uterine segment (from the midpoint to the fundal endometrial apex) and the long axis of the lower uterine segment (from the midpoint to the cervico-corporeal junction).(b)Ultrasonographic image of UCFA measurement in an anteverted uterus with mild intrinsic curvature of the uterine corpus.(c)Ultrasonographic image of UCFA measurement in a patient with severe endometriosis and a positive question mark sign.(d)Schematic diagram of the UFA measurement. (e)Ultrasonographic image of UFA measurement in an anteverted uterus.(f)Ultrasonographic image of UFA measurement in a patient with severe endometriosis.

a

b

d

e

f

c

2.Congenital uterine malformations (e.g., septate uterus, bicornuate uterus, etc.).

3.Intrauterine device in situ, which obscures the midline of the uterine endometrium and may alter the natural curvature of the uterine corpus.

4.Poor image quality, which prevents complete visualization of the uterine corpus and acquisition of a qualified midsagittal plane.
